# Supplementary material for: Endangered Nectar-Feeding Bat Detected by Environmental DNA on Flowers
Source: Animals (Basel). 2022 Nov 8;12(22):3075. doi: 10.3390/ani12223075 (PMC9686659; doi:10.3390/ani12223075)
Supplement: Supplementary file 1 [file animals-12-03075-s001.zip › Table S2.pdf]

Table S2a. Bioinformatics software, parameters, and options used for pre-processing and classifying taxonomy for each of four DNA metabarcoding markers.

				Bats/vertebrates	Plants		Arthropods
**Goal**	**Program**	**Parameter**	**Option**	**SFF-COI**	**ITS2**	**rbcL**	**ANML-COI**
Denoising	DADA2 (Q2)	R1 3' truncation	"--p-trunc-len-f"	202	278	96	175
		R2 3' truncation	"--p-trunc-len-r"	202	240	96	175
		R1 expected errors	"--p-max-ee-f"	2.0	6.0	2.0	2.0
		R2 expected errors	"--p-max-ee-r"	2.0	6.0	2.0	2.0
Singleton ASV removal	feature-table (Q2)	filter-features	"--p-min-frequency"	FALSE	2	2	2
ASV fragment length filtering	feature-table (Q2)	filter-seqs	"--p-where"	202 bp	FALSE	FALSE	FALSE
ITS2 isolation	ITSx	Taxonomic group to retain	"-t"	FALSE	T [higher plants]	FALSE	FALSE
ASV post-clustering	LULU	Min. ratio type	"minimum_ratio_type"	"min"	"min"	"min"	FALSE
		Min. ratio	"minimum_ratio"	0.3	0.3	1.0	FALSE
		Min. match	"minimum_match"	84	84	94	FALSE
		Min. relative cooccurence	"minimum_relative_cooccurenc	0.99	0.99	0.99	FALSE
	Vsearch (Q2)	Percent identity	"--p-perc-identity"	FALSE	FALSE	FALSE	0.985
Relative read abundance (RRA) filtering	R Tidyverse	Relative minimum (Feature/Sample)		FALSE	5%	5%	FALSE
Taxonomic pre-filtering* or classification†	BLAST + MEGAN6	Bitscore	"Min Score"	150†	FALSE	150*	150*
		Min. Percent Identity	"Min Percent Identity"	97†	FALSE	84*	84*
		Top percent	"Top Percent"	5†	FALSE	10*	10*
		Min. support percent	"Min Support Percent"	0.02†	FALSE	0.05*	0.05*
		Query coverage	"Min Percent Read To Cover"	90†	FALSE	89*	89*
		Lowest taxonomic level retained		Species†	FALSE	Kingdom*	Phylum*
		Taxonomy filtered		FALSE	FALSE	Viridiplantae	Arthropoda
Taxonomic classification	naïve-Bayes (Q2)	Training: kmer	"--p-feat-ext--ngram-range"	"[7, 7]"	"[8, 8]"	'"[8, 8]"	"[7, 7]"
		Classification: confidence threshold	"--p-confidence"	0.9	0.6	0.5	0.7
		Reference library		Walker et al. (2016)	Custom (BOLD: Magnoliophyta); PLANitsS attempted (Banchi et al. (2020))	Bell et al. (2017)	O'Rourke et al. (2019)

ITS2 reference library construction and comparison

Toward the taxonomic classification of ITS2 OTUs, initial Naïve Bayes classifier runs using the PLANitS reference library (Banchi et al., 2020) showed coarse taxonomic resolution (classified to family) for OTUs suspected as belonging to the genus *Agave*. Upon further inspection of the reference library, we determined that representative ITS2 sequences corresponding to *Agave* included long homopolymer runs of N characters. We suspected that these homopolymers were lowering our ability to classify *Agave* at higher resolution so we built a new reference library for comparison. On 8 August 2022, we downloaded all available DNA barcode sequences from phylum Magnoliophyta (i.e., flowering plants) from the Barcode of Life Database (Ratnasingham & Hebert, 2007), which included our plant mock community members. We also downloaded all available ITS2 sequences for *Agave havardiana* from NCBI GenBank (Benson et al., 2009). We merged the datasets and retained only ITS2 sequences. We filtered taxonomies to retain sequences with genus-level taxonomies. We then annotated to omit subspecies and variety labels so that the lowest taxonomic rank was species. Following filtering, we retained 71,196 ITS2 sequences. The same way the PLANitS reference library was prepared, we extracted the ITS2 region using ITSx software, a program that uses a hidden Markov model to identify and remove regions flanking ITS2 (Bengtsson-Palme et al., 2013). This yielded a dataset

for extracted ITS2 sequences and a dataset of sequences where the flanking regions were not detected. Upon inspecting non-detections, we determined ITS2 sequences were already extracted when they were submitted so we merged both datasets. Failure to do so would have led to the omission of ~50% of the available sequences. To curate the reference library, we culled and dereplicated using the RESCRIPt software (Robeson et al., 2020) in Qiime2 (Bolyen et al., 2018). For culling, we only retained sequences with a maximum of five degenerate characters and those with homopolymer runs of fewer than 12 homopolymer characters. We dereplicated using the "super" setting. Similar to how the PLANitS reference library was curated, the "super" setting applies a least common ancestor (LCA) consensus to annotate taxonomy labels to a reference library with dereplicated sequences to avoid false positive classification downstream. The general premise of LCA curation is that if a set of matching sequences share contradicting taxonomic labels, the label would be collapsed to the next coarsest taxonomic rank. However, the "super" setting avoids over-collapsing taxonomies and barcode errors by giving preference to majority labels and higher resolution labels (assuming no contradiction). Following dereplication, we retained 43,154 unique taxa-barcode pairs in phylum Magnoliophyta.

A comparison of results for both reference libraries (S2 Table) showed that the BOLD classifier correctly classified the suspected *Agave* features to genus, correctly assigned our six mock community members (3 correctly assigned to species and 3 correctly assigned to genus), and non-*Agave* plant detections were overall consistent with detections made with the PLANitS reference library. Using the same training and classification parameters, the BOLD classifier gave slightly higher species-level recovery, resolving four taxa to species that were also present in the study area. The PLANitS classifier collapsed the same two taxa to genus. Two of the taxa in the BOLD classifier were overclassified to species, whereas the PLANitS correctly collapsed to genus. However, these overclassifications in the BOLD classifier were accounted for by cross-referencing to plants in the study area with the contradicting classifications subsequently collapsed. Our results suggest that the PLANitS reference library is a modestly more careful option when plants in the study area are unknown. However, degenerate characters present in the reference library may lower the resolution of classifications for particular taxa, like *Agave* species. The BOLD reference library, as applied for classification in our study, provides higher recovery to species but may come at the expense of a slightly higher false positive rate at that taxonomic rank. As we did in our study, it is therefore recommended to vet and collapse taxonomic classifications according to local lists of plant species, particularly when using the BOLD reference library that was prepared using the "super" LCA setting in RESCRIPt. Doing so provided largely the same carefulness of classifications but slightly higher species and genus level resolution than classifications from the PLANitS reference library.

Table S2b. Comparison of raw ITS2 taxonomies, prior to relative read abundance filtering and study area cross-referencing, among all samples from classifiers trained with the PLANitS classifier and the BOLD classifier. All available samples for comparison came from the Big Bend study area, whereas ITS2 sequences failed sequencing for the Laguna de Sanchez study area in our pilot testing. An asterisk (*) symbol indicates a species-level classification of a taxon that has been recorded in the study area (Big Bend Study Area). A double cross (‡) indicates a

potential false positive classification at the species-level if the taxon has not been recorded in the study area. A single cross (†) indicates a correct assignment to the taxonomic level classified for a mock community member. Members of the plant mock community included *Bromus carinatus var. marginatus*, *Festuca arizonica*, *Gnaphalium exifolium*, *Houstonia wrightii*, *Juncus saximontanus*, and *Oxalis dillenii.* Note that the taxonomic label for the Agave family is Asparagaceae in the PLANitS reference library, although it is Agavaceae in BOLD.

Sample	PLANitS Classifier (Banchi et al., 2020)	BOLD Classifier (this study)
LENI1	Bouteloua sp.	Bouteloua trifida*
LENI1	Cynodon sp.	Cynodon dactylon*
LENI1	Eragrostis lehmanniana*	Eragrostis lehmanniana*
LENI1	Unassigned	Magnoliopsida sp.
LENI1	Parthenium incanum*	Parthenium sp.
LENI1	Medicago sp.	Medicago sativa*
LENI1	Bouvardia ternifolia*	Bouvardia ternifolia*
LENI1	Oldenlandia ovata‡	Galium sp‡
LENI1	Salvia sp.	Salvia dugesii‡
LENI1	Sphaeralcea sp.	Sphaeralcea munroana‡
LENI1	Oenothera suffrutescens‡	Unassigned
LENI2	Bouteloua curtipendula*	Bouteloua curtipendula*
LENI2	Bouvardia ternifolia*	Bouvardia ternifolia*
LENI3	Sporobolus airoides*	Sporobolus airoides*
LENI3	Unassigned	Magnoliopsida sp.
LENI3	Bouvardia ternifolia*	Bouvardia ternifolia*
LENI3	Tecoma stans*	Tecoma stans*
LENI3	Menodora scabra*	Menodora sp
LENI3	Maurandya antirrhiniflora‡	Maurandya antirrhiniflora‡
LENI3	Sphaeralcea sp.	Sphaeralcea munroana‡
LENI4B	Asparagaceae sp.	Agave sp.
LENI4	Asparagaceae sp.	Agave sp.
LENI5B	Asparagaceae sp.	Agave sp.
LENI5	Asparagaceae sp.	Agave sp.
LENI6B	Asparagaceae sp.	Agave sp.
LENI6B	Muhlenbergia sp.	Muhlenbergia sp.
LENI6B	Oenothera suffrutescens‡	Unassigned
LENI6	Asparagaceae sp.	Agave sp.
LENI7	Asparagaceae sp.	Agave sp.
LENI8	Asparagaceae sp.	Agave sp.
LENI9	Asparagaceae sp.	Agave sp.
LENI9	Sporobolus airoides*	Sporobolus airoides*
LENI9	Bouvardia ternifolia*	Bouvardia ternifolia*

Sample	PLANitS Classifier (Banchi et al., 2020)	BOLD Classifier (this study)
LENI9	Tragia ramosa*	Tragia volubilis``
LENI9	Rhus sp.	Rhus virens*
mock	Juncus nevadensis	Juncus sp.†
mock	Bromus sp.†	Bromus carinatus†
mock	Festuca sp.†	Festuca sp.†
mock	Panicum sp.	Panicum sp.
mock	Gnaphalium exilifolium†	Gnaphalium exilifolium†
mock	Houstonia wrightii†	Houstonia wrightii†

References

Banchi, E., Ametrano, C. G., Greco, S., Stanković, D., Muggia, L., & Pallavicini, A. (2020). PLANiTS: A curated sequence reference dataset for plant ITS DNA metabarcoding. *Database*, *2020*, baz155. https://doi.org/10.1093/database/baz155

Bengtsson-Palme, J., Ryberg, M., Hartmann, M., Branco, S., Wang, Z., Godhe, A., Wit, P. D., Sánchez-García, M., Ebersberger, I., Sousa, F. de, Amend, A., Jumpponen, A., Unterseher, M., Kristiansson, E., Abarenkov, K., Bertrand, Y. J. K., Sanli, K., Eriksson, K. M., Vik, U., … Nilsson, R. H. (2013). Improved software detection and extraction of ITS1 and ITS2 from ribosomal ITS sequences of fungi and other eukaryotes for analysis of environmental sequencing data. *Methods in Ecology and Evolution*, *4*(10), 914–919. https://doi.org/10.1111/2041-210X.12073

Benson, D. A., Karsch-Mizrachi, I., Lipman, D. J., Ostell, J., & Sayers, E. W. (2009). GenBank. *Nucleic Acids Research*, *37*(suppl_1), D26–D31. https://doi.org/10.1093/nar/gkn723

Bolyen, E., Rideout, J. R., Dillon, M. R., Bokulich, N. A., Abnet, C., Al-Ghalith, G. A., Alexander, H., Alm, E. J., Arumugam, M., Asnicar, F., Bai, Y., Bisanz, J. E., Bittinger, K., Brejnrod, A., Brislawn, C. J., Brown, C. T., Callahan, B. J., Caraballo-Rodríguez, A. M., Chase, J., …

Caporaso, J. G. (2018). *QIIME 2: Reproducible, interactive, scalable, and extensible microbiome data science* (e27295v2). PeerJ Inc. https://doi.org/10.7287/peerj.preprints.27295v2

Ratnasingham, S., & Hebert, P. D. N. (2007). bold: The Barcode of Life Data System (http://www.barcodinglife.org). *Molecular Ecology Notes*, *7*(3), 355–364. https://doi.org/10.1111/j.1471-8286.2007.01678.x

Robeson, M. S., O'Rourke, D. R., Kaehler, B. D., Ziemski, M., Dillon, M. R., Foster, J. T., & Bokulich, N. A. (2020). RESCRIPt: Reproducible sequence taxonomy reference database management for the masses. *BioRxiv*, 2020.10.05.326504. https://doi.org/10.1101/2020.10.05.326504
