# Supplementary figures and images for: Endangered Nectar-Feeding Bat Detected by Environmental DNA on Flowers
Source: Animals (Basel). 2022 Nov 8;12(22):3075. doi: 10.3390/ani12223075 (PMC9686659; doi:10.3390/ani12223075)

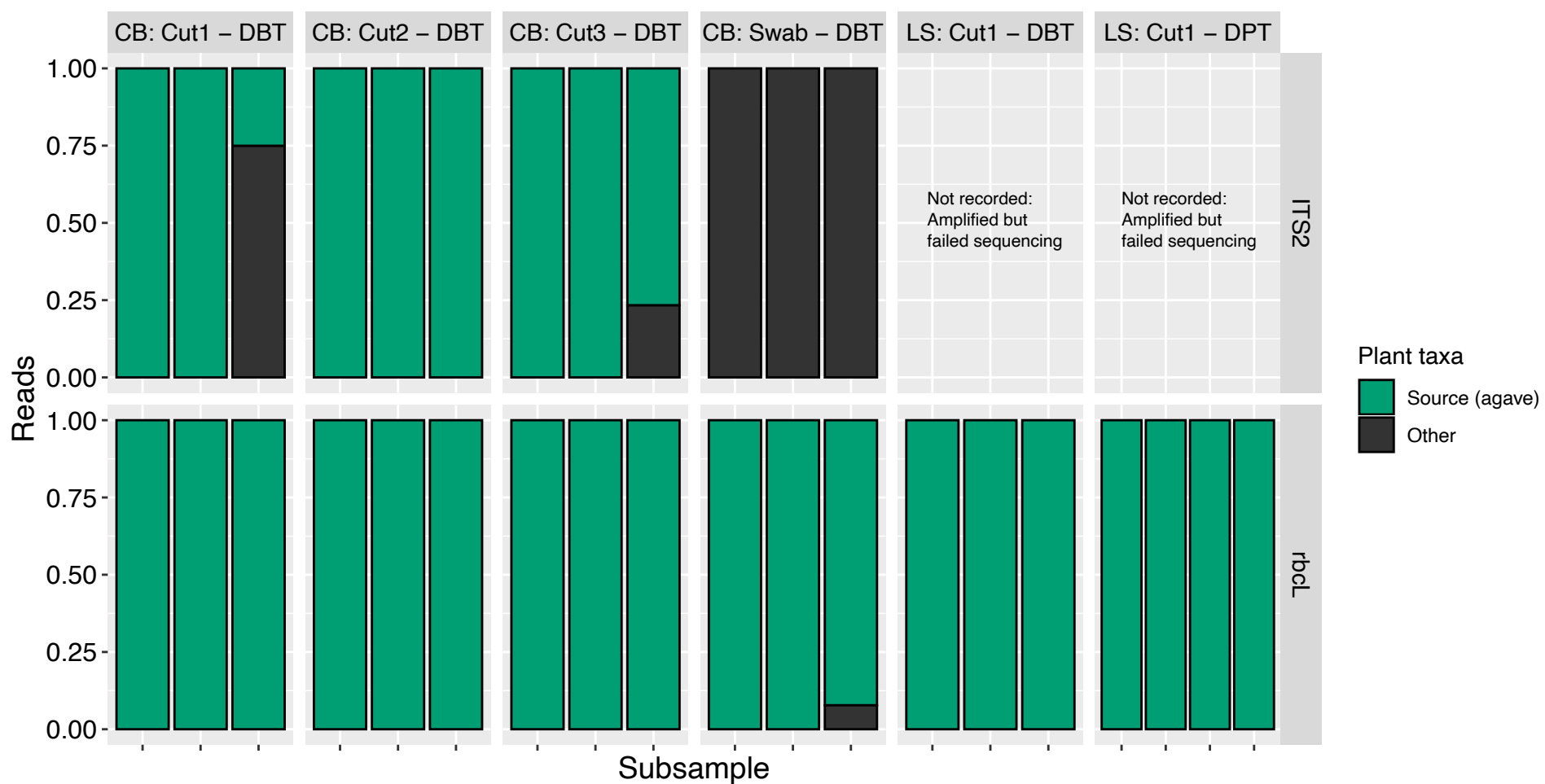

Supplement: Supplementary file 1 [file animals-12-03075-s001.zip › Figure S1.pdf]
